# Supplementary figures and images for: Function-Based Rhizosphere Assembly along a Gradient of Desiccation in the Former Aral Sea
Source: mSystems. 2022 Nov 15;7(6):e00739-22. doi: 10.1128/msystems.00739-22 (PMC9765073; doi:10.1128/msystems.00739-22)

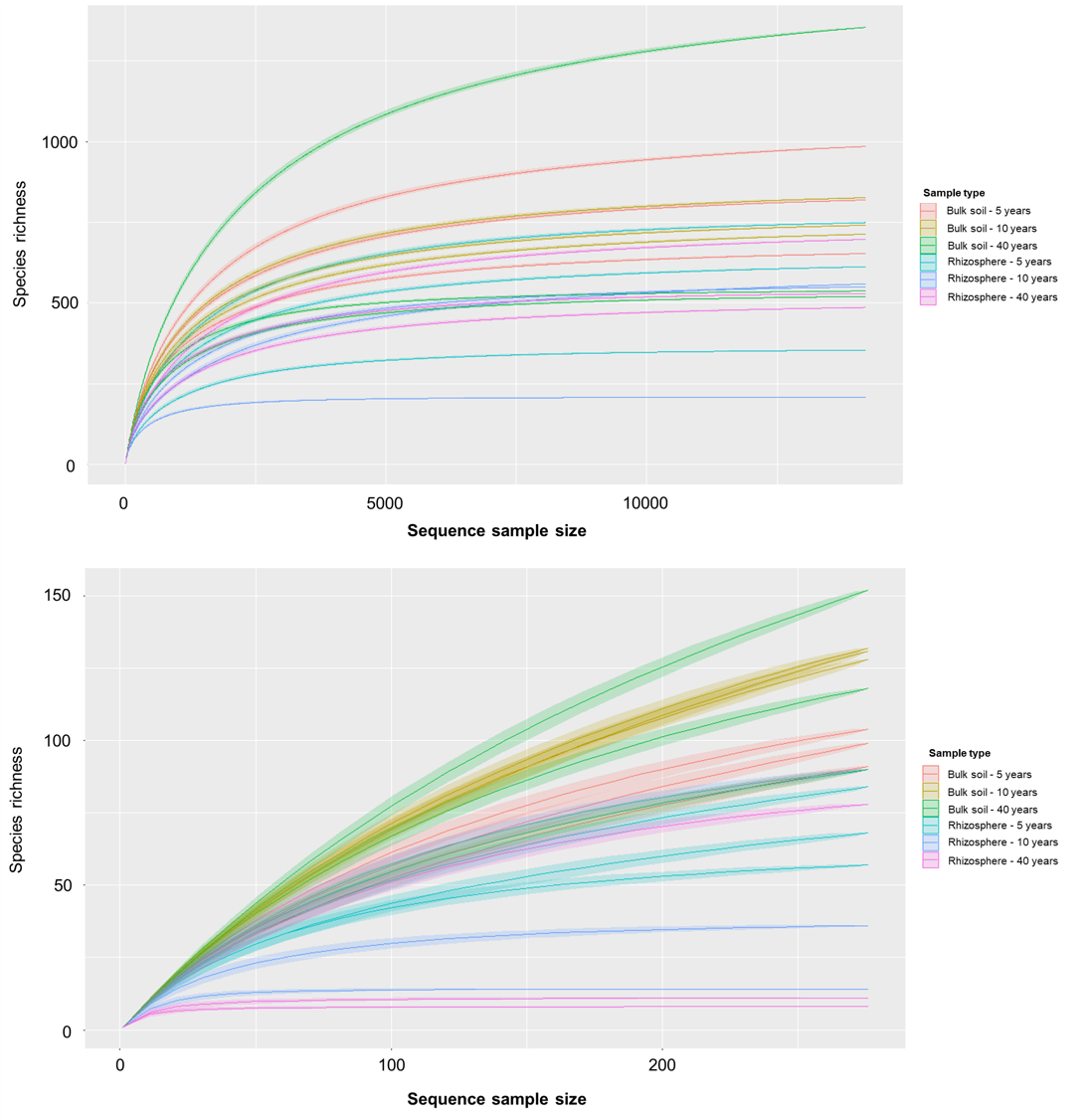

Supplement: FIG S3 [file msystems.00739-22-s0003.tif]

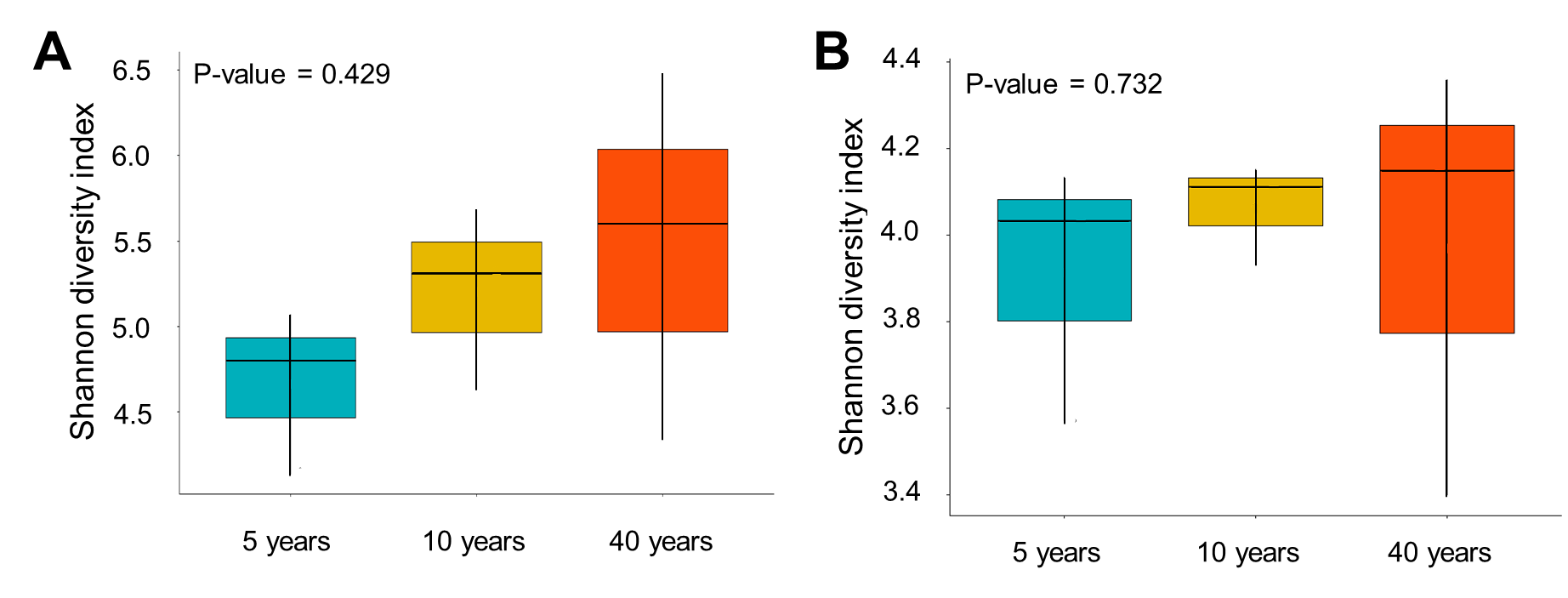

Supplement: FIG S4 [file msystems.00739-22-s0004.tif]

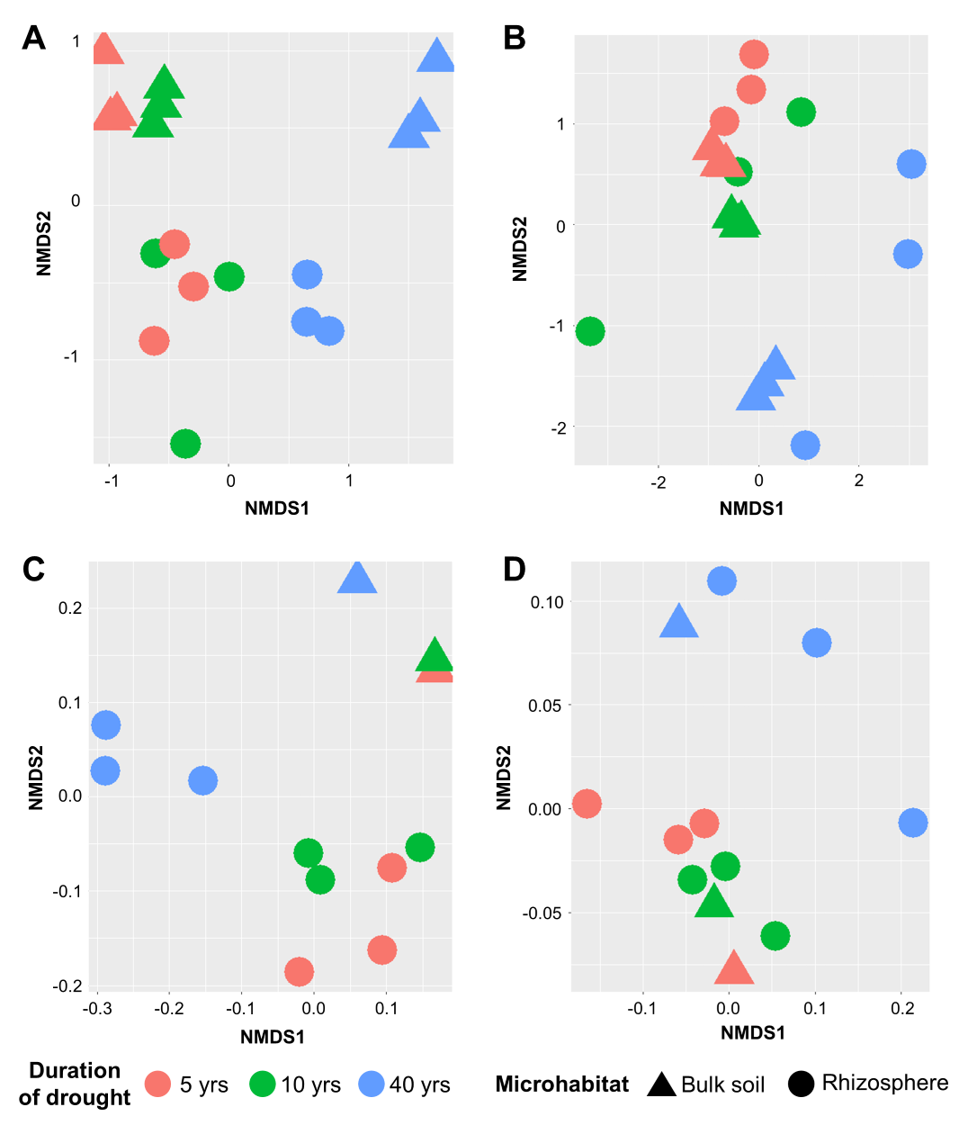

Supplement: FIG S5 [file msystems.00739-22-s0005.tif]

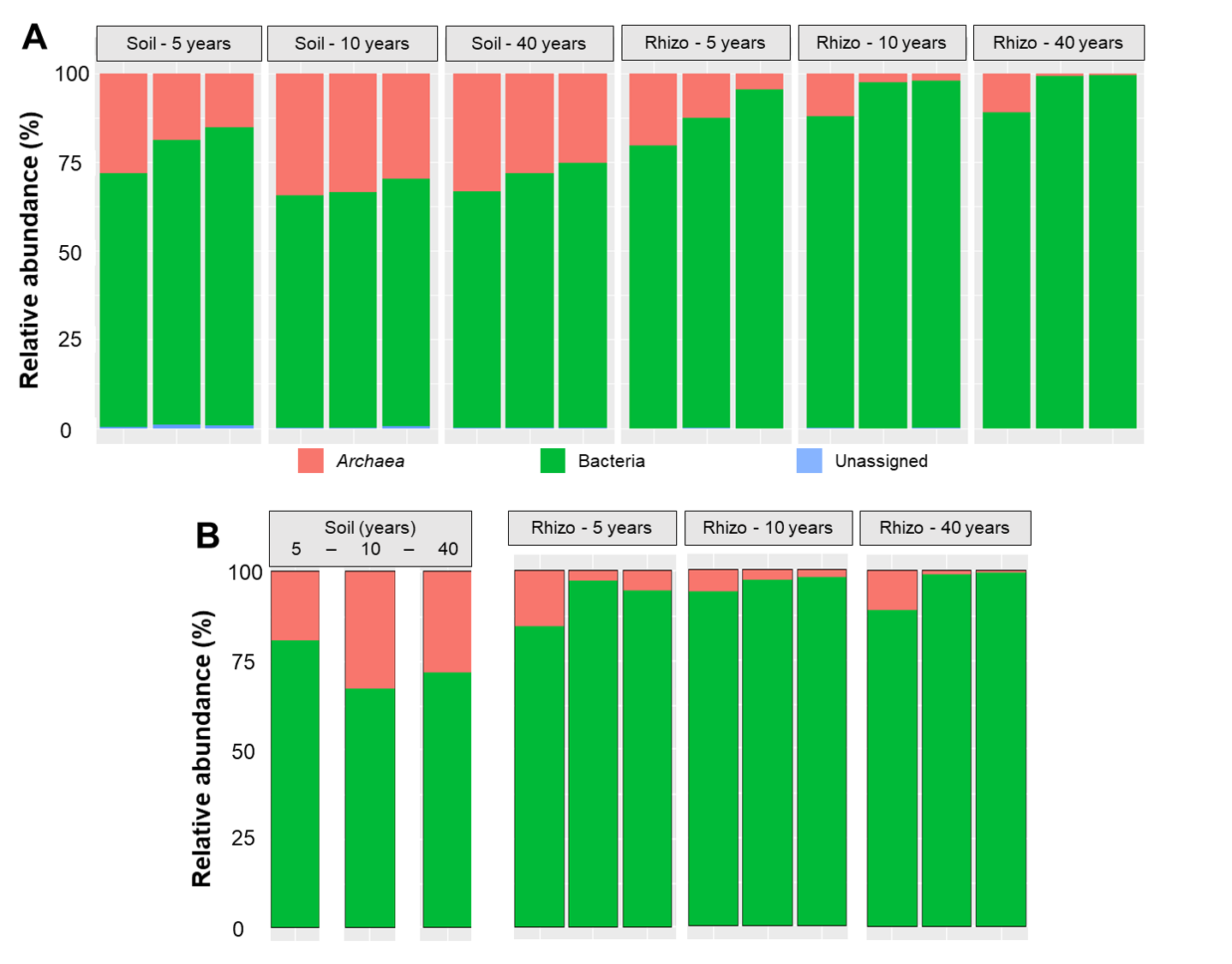

Supplement: FIG S2 [file msystems.00739-22-s0002.tif]

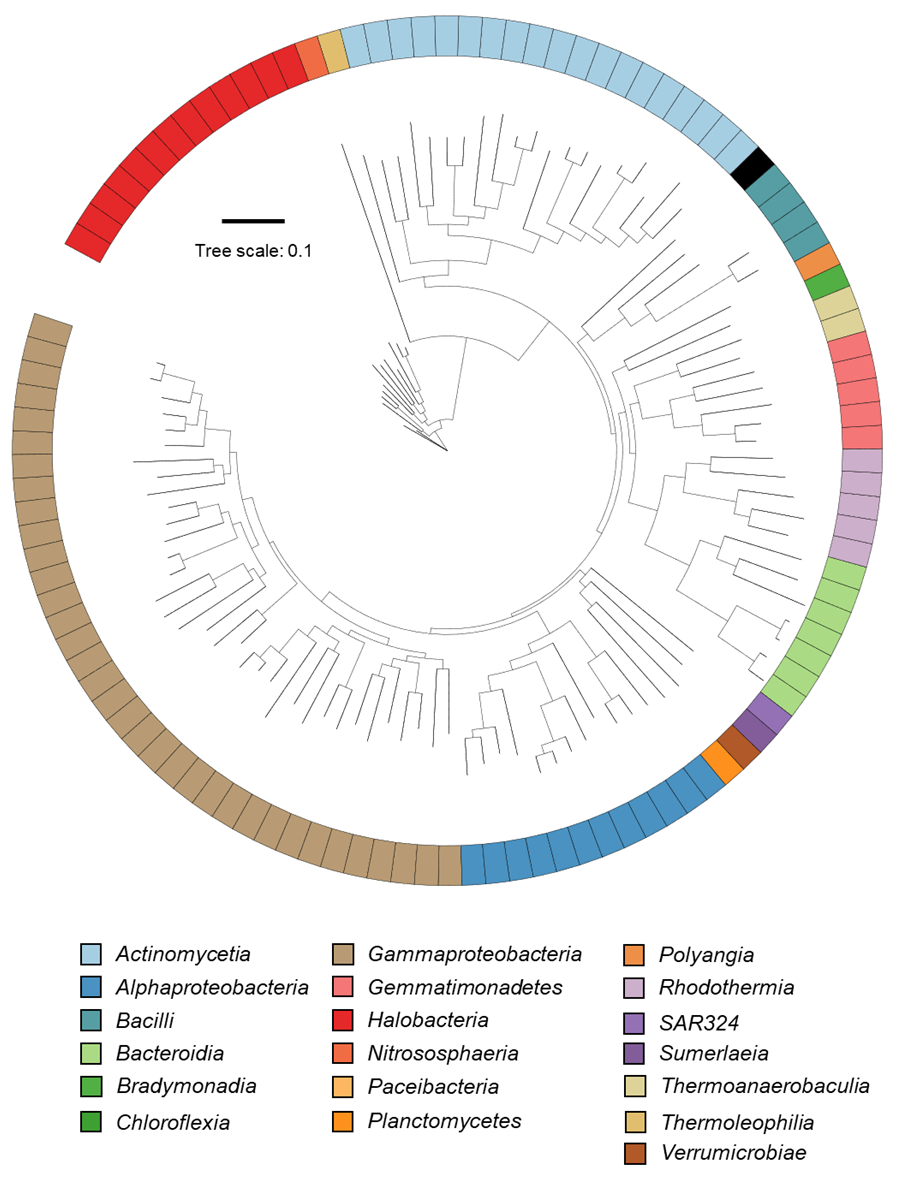

Supplement: FIG S6 [file msystems.00739-22-s0006.tif]

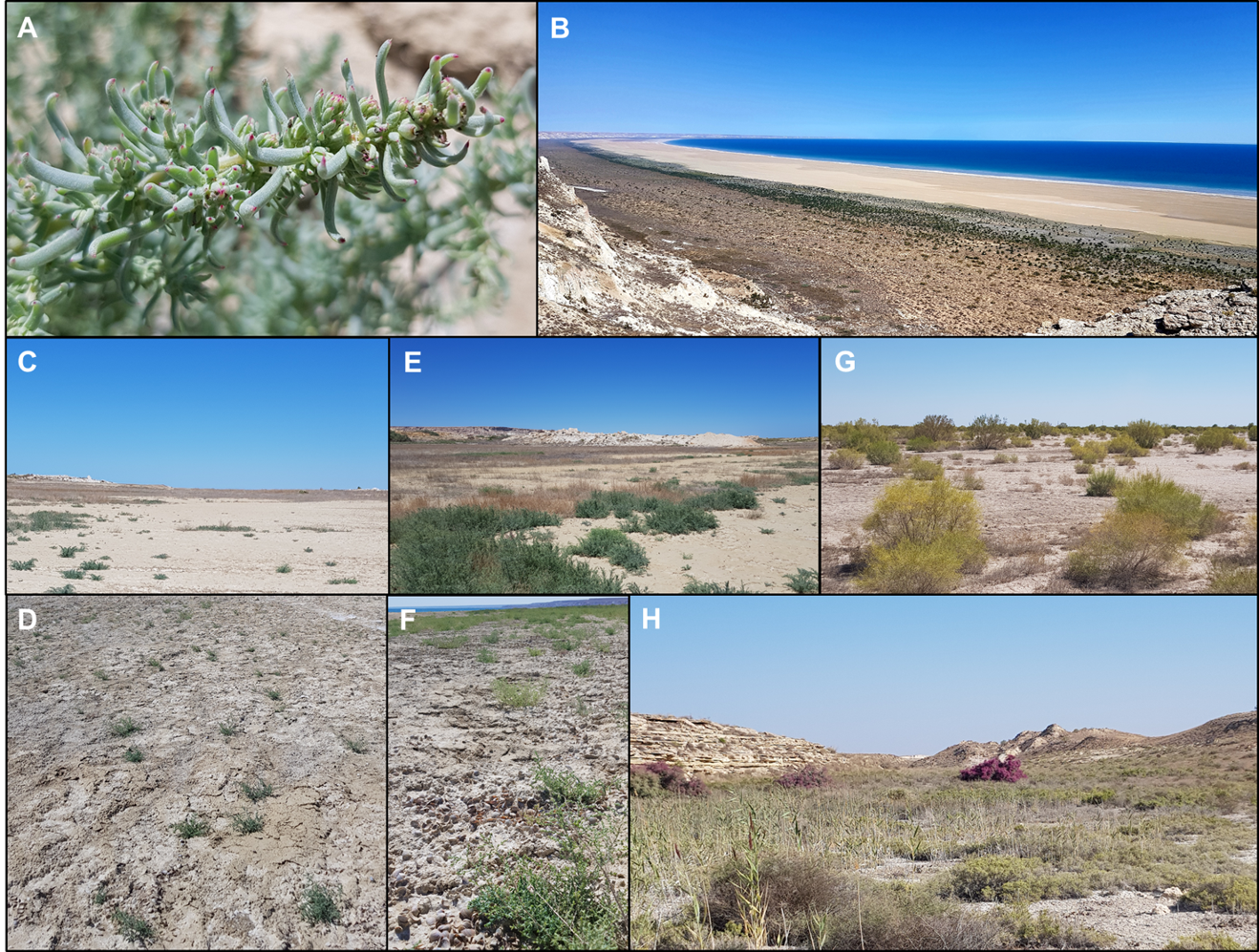

Supplement: FIG S1 [file msystems.00739-22-s0001.tif]
