# Supplementary material for: Function-Based Rhizosphere Assembly along a Gradient of Desiccation in the Former Aral Sea
Source: mSystems. 2022 Nov 15;7(6):e00739-22. doi: 10.1128/msystems.00739-22 (PMC9765073; doi:10.1128/msystems.00739-22)
Supplement: TABLE S2 [file msystems.00739-22-s0008.docx]

**Supplementary Table S2.** Pairwise comparison of bacterial and archaeal diversity based on amplicon sequencing data between bulk soil and rhizosphere subjected to different periods of drought using Dunn's test

| Dataset | Comparison | *P* value |
| --- | --- | --- |
| Bacteria | Soil_year_05 - Rhizopshere_year_05 | 0.047* |
|  | Soil_year_10 - Rhizopshere_year_10 | 0.009* |
|  | Soil_year_40 - Rhizopshere_year_40 | 0.193 |
| Archaea | Soil_year_05 - Rhizopshere_year_05 | 0.251 |
|  | Soil_year_10 - Rhizopshere_year_10 | 0.022* |
|  | Soil_year_40 - Rhizopshere_year_40 | 0.014* |

*Asterisk indicates a significance different in bacterial/archaeal diversity according to the Dunn's test pairwise comparison
