# Supplementary material for: Function-Based Rhizosphere Assembly along a Gradient of Desiccation in the Former Aral Sea
Source: mSystems. 2022 Nov 15;7(6):e00739-22. doi: 10.1128/msystems.00739-22 (PMC9765073; doi:10.1128/msystems.00739-22)
Supplement: TABLE S3 [file msystems.00739-22-s0009.docx]

**Supplementary Table S4.** PERMANOVA-based assessments of factors that significantly influenced bacterial and archaeal communities

| Taxa | Sequencing approach | Factors | R^2^ value | *P* value |
| --- | --- | --- | --- | --- |
| Bacteria | 16S amplicon | Microhabitat | 0.164 | 0.001 |
|  |  | Years without water | 0.242 | 0.001 |
|  |  | Microhabitat x Years without water | 0.173 | 0.007 |
|  | Shotgun metagenome | Years without water | 0.658 | 0.008 |
| Archaea | 16S amplicon | Microhabitat | 0.108 | 0.001 |
|  |  | Years without water | 0.246 | 0.001 |
|  |  | Microhabitat x Years without water | 0.151 | 0.007 |
|  | Shotgun metagenome | Years without water | 0.499 | 0.003 |
