# Supplementary material for: Function-Based Rhizosphere Assembly along a Gradient of Desiccation in the Former Aral Sea
Source: mSystems. 2022 Nov 15;7(6):e00739-22. doi: 10.1128/msystems.00739-22 (PMC9765073; doi:10.1128/msystems.00739-22)
Supplement: TABLE S1 [file msystems.00739-22-s0007.docx]

**Supplementary Table S1.** Details of the amplicon and shotgun metagenome sequencing of different samples from the dried-out Aral Sea basin

| Sample ID | Sample type | Years without water | Number of bacterial reads according to amplicon sequencing | Number of archaeal reads according to amplicon sequencing | Number of bacterial reads according to Kraken2 | Number of archaeal reads according to Kraken2 | Number of bacterial reads according to eggNOG mapper | Number of archaeal reads according to eggNOG mapper |
| --- | --- | --- | --- | --- | --- | --- | --- | --- |
| AS1 | Rhizosphere | 5 | 34016 | 9,935 | 4.2E+07 | 7,509,242 | 25401899 | 3,461,634 |
| AS2 | Rhizosphere | 5 | 65594 | 3,502 | 3.8E+07 | 977,465 | 26755717 | 665,848 |
| AS3 | Rhizosphere | 5 | 53836 | 9,466 | 3.7E+07 | 2,083,068 | 26278557 | 1,439,207 |
| AS4 | Rhizosphere | 10 | 51348 | 7,714 | 3.4E+07 | 2,087,431 | 32428407 | 1,669,808 |
| AS5 | Rhizosphere | 10 | 14206 | 465 | 3.6E+07 | 949,513 | 24603727 | 784,341 |
| AS6 | Rhizosphere | 10 | 74906 | 1,679 | 2.5E+07 | 473,215 | 22341460 | 483,126 |
| AS7 | Rhizosphere | 40 | 48523 | 6,643 | 1.4E+07 | 1,692,174 | 11659085 | 979,208 |
| AS8 | Rhizosphere | 40 | 57060 | 276 | 3.6E+07 | 161,080 | 20305790 | 162,598 |
| AS9 | Rhizosphere | 40 | 62051 | 440 | 2.8E+07 | 68,931 | 16080548 | 124,704 |
| AS10 | Soil | 5 | 48347 | 19,394 | 1.1E+07 | 2,601,462* | 18912120 | 2,664,479* |
| AS10_1 | Soil | 5 | 53238 | 9,751 |  |  |  |  |
| AS10_2 | Soil | 5 | 55076 | 13,113 |  |  |  |  |
| AS11 | Soil | 10 | 56888 | 29,270 | 7099087 | 3,436,544* | 12481075 | 2,754,117* |
| AS11_1 | Soil | 10 | 53996 | 23,422 |  |  |  |  |
| AS11_2 | Soil | 10 | 59606 | 31,735 |  |  |  |  |
| AS12 | Soil | 40 | 86529 | 34,893 | 1.2E+07 | 4,866,227* | 10783281 | 3,490,074* |
| AS12_1 | Soil | 40 | 35519 | 18,382 |  |  |  |  |
| AS12_2 | Soil | 40 | 40936 | 14,654 |  |  |  |  |

* The value is based on pooled samples of the triplicates
